# Kreosote a Remedy for Vomiting

**Published:** 1847-09

**Authors:** 


*Kreosote a remedy for Vomiting.*—As vomiting is apt to be a troublesome symptom in the diseases of the summer season, we would invite the attention of our professional brethren to the above remedy. We have employed it very considerably in our practice for the last three years, and in a large proportion of cases, proceeding from gastric irritation, both in children and adults, it has had an efficacy so immediate and complete, as almost to entitle it to be considered a specific. We recommend it in Cholera Infantum, and in common Cholera Morbus, in so far as vomiting is an element of these affections—and, indeed, in all cases of vomiting, not involving gastric inflammation—with confidence that it will be found an invaluable remedy.

The following is our method of prescribing it for adults, and for children over two years of age. For children under two years, we generally diminish the quantity of kreosote one half.

℞. Kreosote gtt. viii.

P. Sacch. alb. ʒi.

Spts. Lav. comp. ʒii.

Aquæ Pur. ℥vi.

f. Mist.

A teaspoonful to be administered directly after each act of vomiting. In a good proportion of cases one dose only is requisite.

